# *Helicobacter pylori* Eradication Lowers Serum Asymmetric Dimethylarginine Levels

**DOI:** 10.1155/2010/685903

**Published:** 2010-12-13

**Authors:** Selim Aydemir, Hacı Eren, Ishak Ozel Tekin, Ferda Akbay Harmandar, Nejat Demircan, Mehmet Cabuk

**Affiliations:** ^1^Department of Gastroenterology, Faculty of Medicine, Zonguldak Karaelmas University, 67800 Zonguldak, Turkey; ^2^Department of Internal Medicine, Faculty of Medicine, Zonguldak Karaelmas University, 67800 Zonguldak, Turkey; ^3^Department of Immunology, Faculty of Medicine, Zonguldak Karaelmas University, 67800 Zonguldak, Turkey; ^4^Department of Family Medicine, Faculty of Medicine, Zonguldak Karaelmas University, 67800 Zonguldak, Turkey; ^5^Department of Nuclear Medicine, Faculty of Medicine, Zonguldak Karaelmas University, 67800 Zonguldak, Turkey

## Abstract

*Introduction*. Microbial pathogens, one of them is *Helicobacter pylori* (*H. pylori*), have frequently been implicated in the atherogenesis. Endothelium-derived nitric oxide (NO) is synthesized from L-arginine by nitric oxide synthase (NOS) and plays a pivotal role in the regulation of vascular tone. Asymmetric dimethylarginine (ADMA) is the most potent endogenous NOS inhibitor. Elevated levels of ADMA have been reported in many circumstances associated with a high cardiovascular risk. The aim of the present study was to investigate whether the eradication of *H. pylori* infection affects serum ADMA levels. *Materials and Methods*. Forty-two *H. pylori*-positive patients were enrolled in the study. Triple therapy for 14 days were given to all patients. Serum ADMA levels were measured at baseline and 2 months after therapy. *Results*. Eradication was achieved in 34 (81%) patients. The mean serum ADMA levels before and after therapy were 1, 77 ± 0, 30 and 1, 67 ± 0, 29 ng/mL in the group with *H. pylori* eradicated and 1, 63 ± 0, 28 and 1, 56 ± 0, 32 ng/mL in the noneradicated, respectively. We detected statistically significant decreased serum ADMA levels after therapy in *H. pylori* eradicated group. *Conclusion*. These findings have indicated that eradication of *H. pylori* infection may decrease the risk of atherosclerosis and cardiovascular events.

## 1. Introduction

Immunoinflammatory events due to chronic infection are thought to be one of the definitive atherogenetic processes [1]. Evidences in favor of this hypothesis are derived from in-vitro experimental and seroepidemiologic studies [2, 3].

*Helicobacter pylori (H. pylori) is* the most frequently encountered bacterial infection worldwide [4]. It is localized within the gastric mucosa leading to life-long inflammation [4, 5]. *H. pylori has* been accused in the pathogenesis of many diseases, either digestive or nondigestive type. One of the most controversial nongastric diseases for that *H. pylori *held responsible is coronary heart disease [6–10].

 Nitric oxide (NO) is synthesized from L-arginine by nitric oxide synthase (NOS) [11] and is a substance that plays an important role in vascular homeostasis regulating vessel tonus [12]. At the same time, it counteracts some vital steps in atherosclerosis such as platelet aggregation, leukocyte-endothelium interaction, and proliferation of vascular smooth muscle cells [12, 13]. The evidences derived from in vivo and in vitro studies give rise to think NO is a decisive regulatory molecule in atherogenesis and arteriogenesis that are major processes for the formation of collateral arteries [14–17].

Asymmetric dimethylarginine (ADMA) is an endogenous competitive inhibitor of endothelial NOS and decreases its production and bioavailability [18]. ADMA is produced by methylation of arginine remnants during normal protein cycle in many tissues including vascular endothelium [12]. It is metabolized to citrulline via dimethylaminohydrolase [12, 19]. 

 A lot of data have recently been published on the importance of ADMA in endothelial dysfunction as a cardiovascular risk factor [20–23].

In the literature, there are a very few number of studies on serum ADMA levels in *H. pylori* infections and those studies reveal somehow controversial results [11, 20]. The effects of *H. pylori* eradication on serum ADMA levels have not been evaluated recently. In the present study, we were interested to see the effects of *H. pylori *eradication on serum ADMA levels.

## 2. Methods

### 2.1. Patients

Forty-two patients with dyspeptic symptoms were enrolled into the study. Exclusion criteria were prior eradication therapy for *H. pylori*, antiulcer drug use within last 1 month, gastrointestinal system and other organ malignancies, inflammatory and infectious diseases, and prior gastric surgery. Patients with chronic diseases such as ischemic heart disease, diabetes mellitus, or hypertension were not included in the study. The state of *H. Pylori *infection was diagnosed by ^14^C-urea breath test.

Eradication therapies consisted of lansoprazole 30 mg (2 × 1/day), clarithromycin 500 mg (2 × 1/day), and amoxicillin 1000 mg (2 × 1/day) taken for 2 weeks and used in all participants. 

In all the patients, eradication was verified by means of ^14^C-urea breath test 2 months after *H. Pylori *eradication treatment.

This study was conducted with the permission of the local ethics committee of Zonguldak Karaelmas University Faculty of Medicine, Zonguldak, Turkey.

Fasting blood samples of the subjects were drawn for the analysis before and 2 months after *H. Pylori *eradication treatment. The blood samples were kept at room temperature for 30 min, and then sera were separated from the cells by centrifugation at 3000 rpm for 5 min. Serum samples were stored at −80°C until the day of analysis.

### 2.2. Measurement of ADMA Levels

Serum ADMA levels were measured by a commercially available kit (Immun Diagnostik AG, Bensheim, Germany) based on enzyme-linked immunosorbent assay (ELISA) method.

### 2.3. Statistical Analysis

Statistical analyses were performed using the statistical package for the social sciences (SPSS, Version 13.0). Results were expressed as the means ± standard deviations. In the comparison between groups, statistically significant differences were assessed by the Wilcoxon signed ranks test or paired *t* tests. *P* < .05 was considered statistically significant.

## 3. Results

Forty-two patients with dyspeptic symptoms (20 males, 22 females) with mean age of 39,1 ± 10,6 years (min 19, max 54) were enrolled into the study.

Eradication was achieved in 34 (81%) patients. The mean serum ADMA levels before and after therapy were 1,77 ±  0, 30 and 1,67 ± 0, 29 ng/mL in the group with *H. pylori *eradicated and 1,63 ± 0, 28 and 1,56 ±  0, 32 ng/mL in the noneradicated, respectively. We could detect statistically significant decrease in serum ADMA levels after *H. pylori* therapy in *H. pylori* eradicated group (*P* < .05; Table 1).

## 4. Discussion

*H. pylori* is a known causal agent of several gastrointestinal diseases and has also been implicated in ischemic heart disease. However, the role of *H. Pylori *in the pathogenesis of atherosclerosis is controversial and the mechanism in this association is also unclear. Several different mechanisms are proposed for the role of *H. pylori* in ischemic heart disease [1–4, 9, 10, 24]. 

In the present study, we investigated to see the effects of *H. pylori *eradication on serum ADMA levels, an NOS inhibitor. The analysis showed that *H. Pylori *eradication was associated with a significant reduction in serum ADMA levels. In the literature, these are the first data that have shown the effects of *H. pylori *eradication therapy on serum ADMA levels. 

Nitric oxide is a substance derived from an aminoacid, L-arginine, by an endothelial NOS enzyme and plays a crucial role in maintaining vascular homeostasis by regulating vessel tonus [12]. 

ADMA is a naturally occurring amino acid that has the property of inhibiting the activity of NOS [25]. ADMA is widely present throughout the body [11]. ADMA is the most potent endogenous NOS inhibitor [25]. 

Currently available experimental and clinical evidence suggests that even small modifications of circulating ADMA levels significantly change vascular NO production, vascular tone, and systemic vascular resistance [26, 27].

Elevated ADMA levels have been detected in a large number of diseases associated with an impaired endothelial L-arginine-NO pathway, such as atherosclerosis, hypercholesterolemia, chronic heart failure, type 2 diabetes mellitus, stroke, hyperhomocysteinemia, and hypertension [25, 26, 28]. Moreover, ADMA has recently been shown to be a risk factor for cardiovascular disease [29, 30]. 

*H. pylori *which is a spiral-shaped gram-negative microorganism causes one of the most prevalent chronic infections worldwide [4]. *H. pylori *typically leads to a life-long chronic infection in the stomach after exposure. *H. pylori *first colonizes on the gastric epithelium within the mucous layer. Then, it causes active chronic inflammation which is composed of gastric submucosal neutrophilic and monocytic infiltration. *H. pylori *plays a proinflammatory role by inducing various gastric mucosal mediators regulating the motion of neutrophils and other leukocytes, which leads to gastric mucosal damage and epithelial remodeling [6, 31, 32]. 

The reason why ADMA increased in *H. pylori* is not clear. The production of endogenous ADMA is influenced by many factors. Proinflammatory factors could induce oxidative stress to increase ADMA level in cardiovascular tissues via reduction of dimethylarginine dimethylaminohydrolase (DDAH) activity that degrades ADMA [11, 33–36]. In addition, TNF-alpha increased ADMA in endothelial cells [37]. *H. pylori* infection was proven that oxidative stress [38–40] and cytokine levels like TNF-alpha [40, 41] changed in chronic inflammation secondary to inflammatory cell proliferation in gastric mucosa. Those changes of cytokine levels and oxidative stress in *H. pylori *infection might cause ADMA level elevations. Also *H. pylori *infection might itself produce ADMA [20, 42]. Furthermore, chronic *H. pylori *infection might decrease the absorption of micronutrients, hence, causing an imbalance in homocysteine-methionine system, so increasing methyl reservoir and then ADMA levels [20, 43].

Very few studies have been found in the literature stressing on ADMA levels in *H. pylori* infection. Marra et al. found that ADMA levels increased in *H. pylori-*positive case group compared to negative control [20]. They suggested that eradication of *H. pylori *might decrease the risk of cardiovascular disease by decreasing serum ADMA levels. However, they did not investigate the effect of *H. pylori *eradication on the ADMA levels. 

In another study conducted by Zhang et al., they found ADMA at high levels in gastric juice of *H. pylori-*positive cases whereas did not encounter any difference between *H. pylori-*positive and negative patients with respect to plasma ADMA levels [11]. 

In the present study different from the previous ones we focused that *H. pylori *eradication significantly decreased the serum ADMA levels. We have postulated that *H. pylori* eradication might be important in preventing the diseases, for example, cardiovascular diseases, related to ADMA by decreasing its serum levels.

## Figures and Tables

**Table 1:** Pretreatment and posttreatment ADMA levels.

	ADMA pretreatment (ng/mL)	ADMA posttreatment (ng/mL)	*P*
*H. pylori *eradicated group	1,77 ± 0, 30	1,67 ± 0, 29	<.05
*H. pylori *non-eradicated group	1,63 ± 0, 28	1,56 ± 0, 32	NS

NS: Not significant.
